# Electromyographic activity of pelvic and lower limb muscles during postural tasks in people with benign joint hypermobility syndrome and non hypermobile people. A pilot study

**DOI:** 10.1016/j.math.2011.07.005

**Published:** 2011-12

**Authors:** Naomi L. Greenwood, Lynsey D. Duffell, Caroline M. Alexander, Alison H. McGregor

**Affiliations:** a Human Performance Group, Division of Surgery, Department of Surgery and Cancer, Faculty of Medicine, Imperial College London, London W6 8RF, United Kingdom; b Department of Physiotherapy, Imperial College Healthcare NHS Trust, Charing Cross Hosptial, Fulham Palace Road, London W6 8RF, United Kingdom

**Keywords:** Electromyography, Hypermobility, Muscle, Posture

## Abstract

Benign joint hypermobility syndrome (BJHS) is associated with the early development of certain degenerative conditions, which may be associated with altered muscle activity. This pilot study compared muscle activation patterns during postural tasks between people with BJHS who do not have pain and people with normal flexibility (control group). Sixteen subjects aged 22–45 years (8 with BJHS) were selected from a population recruited to a larger study. Electromyographic activity of erector spinae (ES), gluteus medius (GM), and lower limb (rectus femoris (RF), semitendinosus (ST), tibialis anterior (TA) and gastrocnemius lateralis) muscles was assessed, and chosen based on the muscles being tested in the larger study. Subjects carried out 30 s of quiet standing (QS) and one-leg standing (OLS), both with eyes open (EO) and eyes closed (EC). Both groups had significantly more TA activity, and control subjects had significantly more GM activity, during OLS EC compared with QS. GM activity was not significantly different between groups. Compared with the BJHS group, control subjects had significantly less ST activation overall, significantly more ES activity during OLS EC and significantly less RF-ST co-contraction during QS. This study has noted differences in muscle activation patterns between pain-free hypermobile people and control subjects, specifically involving muscles surrounding the pelvis and hip. This pilot data suggests that strategies for stabilising the body during balancing tasks may be relevant to injury risk in people with BJHS. While results need to be verified with a larger subject sample, this study is important in developing new treatments for hypermobile people.

## 1 Introduction

Benign joint hypermobility syndrome (BJHS) is a hereditable collagen disorder that features excessive flexibility of joints and chronic pain. It is closely associated with a genetic disorder, the hypermobile type of Ehlers Danlos Syndrome (EDS type III) (Grahame, 2008). Previously this condition was considered an insignificant finding due in part to the absence of any non-musculoskeletal symptoms. However over the past few decades, research into the area revealed important findings that link this symptom to more serious conditions such as osteoarthritis (OA) (Bridges et al., 1992) or low back pain (LBP) (Murray, 2006).

An abnormality of structure and distribution of type I collagen together with an increased ratio of collagen type III to type I is thought to be the underlying cause of BJHS (Russek, 1999), resulting in decreased stiffness and generalised ligament laxity, which constitutes the clinical picture observed in patients.

The effect of joint laxity ranges from joint pain to increased soft tissue injuries and joint subluxation or dislocation. Extra-articular manifestations may include mitral valve prolapse, which is three times more prevalent in BJHS populations than healthy populations, uterine and rectal prolapse and abdominal herniation. Other associations include increased incidence of anxiety disorders and delayed motor development in infants (Grahame, 1990).

A diagnosis of BJHS may bring with it an increased risk of developing degenerative diseases such as OA (Grahame, 1989; Bridges et al., 1992; Jonsson et al., 1996); one study reported that up to 60% of BJHS patients developed OA (Bridges et al., 1992). Investigations have found reduced proprioception in both BJHS (Mallik et al., 1994) and OA (Hassan et al., 2001) subjects compared to healthy controls, as well as an association between injury risk and core proprioception in athletes (Zazulak et al., 2007). These findings have highlighted the significance of reduced proprioception and how it may contribute to disease progression. Proprioception involves a complex interplay between central processing, peripheral proprioceptive receptors and the activation of specific muscles (Hassan et al., 2001). It is a vital feedback mechanism that allows the body to perceive where limbs are positioned and initiates appropriate muscle recruitment to ensure posture is maintained.

It has been suggested that the defect in collagen and resulting ligament laxity not only increases the range of movement of a joint, but leads to the adoption of hyperextended postures as a result of decreased stability (Hall et al., 1995). It could be speculated that the resultant repeated trauma and wear from these abnormal postures may be the cause of increased OA incidence within the BJHS population.

Treatment options for BJHS patients have been given little attention and, as a result, patients are often left untreated. Physiotherapy as a treatment has been explored with some success. The aim of such treatments is to strengthen supporting muscles, which is thought to increase proprioceptive acuity. The idea comes from the observation that BJHS is widely seen in ballet dancers (Klemp et al., 1984), yet proprioception does not appear to be effected (Barrack et al., 1984). Both treatment and research in BJHS has to date focussed on the structures immediately surrounding the affected joint. However the thorax, trunk and lower limbs are a dynamic structure, and should be treated as such rather than considering each joint in isolation. Recently, the spine has been modelled as an inverted pendulum supported by a moving base (the lower limbs) (McGregor and Hukins, 2009). This model can be extended to suggest that the hip, knee and ankle joints are also moving bases that support the back, upper leg and lower leg respectively. It is thought that problems at a specific joint could be the result of problems that lie elsewhere in this dynamic structure. Indeed, injury risk in sports participants has been associated with both lumbopelvic movement control (Roussel et al., 2009) and core proprioception (Zazulak et al., 2007), and this might explain how instabilities at joints lead to musculoskeletal injuries and conditions such as LBP and OA.

Recently specific attention has been given to the hip musculature; specifically gluteus medius in people with osteoarthritis affecting their knee joint (Chang et al., 2005; Henriksen et al., 2009). It has been proposed that weakness in GM results in contralateral pelvic drop in these subjects and increased loading on the medial knee joint (Chang et al., 2005). We hypothesised that people with BJHS would show differences in the activation of muscles in the leg (quadriceps and hamstrings), which are similar to those seen in people with knee OA, and that these would be associated with changes in muscles that are important in pelvic control (gluteus medius and erector spinae). This may provide important information to inform interventions for people with BJHS.

In this study we investigated the muscle activity within a hypermobile group compared to a healthy control group during postural and balance tasks. We hypothesised that BJHS leads to altered recruitment patterns in muscles of both the pelvis and the lower limbs.

## 2 Methods

### 2.1 Participants

Subjects were recruited through email advertising within the Physiotherapy, Occupational Heath and Dietetics departments at the Imperial College Heathcare NHS Trust. Further recruitment involved email advertising within the author’s university year group and research colleagues. Ethical approval was obtained from the Imperial College Ethics Committee.

Subjects were drawn from a larger study of individuals with and without knee osteoarthritis. The criteria to be included in the present study were healthy people aged between 18 and 50 years who had no clinical or radiological symptoms of knee osteoarthritis and who can walk without the use of an assistive device. The exclusion criteria were any neurological or painful musculoskeletal conditions involving the lower limbs, rheumatoid or any other systemic arthritis and obesity (Body Mass Index (BMI) >35).

A total of 16 subjects (4 male and 12 female) were recruited with an average age of 28 years (range 22–45 years). Eight subjects (3 male) had BJHS and 8 subjects (1 male) were controls. Average height (SD), weight and BMI of the hypermobile *vs* control subjects were 1.6 (0.1) *vs* 1.7 (0.1) m, 64.8 (5.4) *vs* 68.6 (9.5) kg and 22.6 (1.4) *vs* 23.5 (3.7), respectively. There were no significant differences between groups for these parameters. Both hypermobile and control subjects were free from pain at the time of testing, and had no history of back pain.

### 2.2 Assessment of benign joint hypermobility syndrome

The Beighton Criteria (Beighton et al., 1973) was used to determine whether the subjects were considered hypermobile. Subjects were shown the movements that make up the Beighton criteria and asked to reproduce them. One point was awarded for each of the nine movements that were re-produced. A score of 4 or greater was considered hypermobile for the purpose of this study.

Eight of the subjects were hypermobile with an average score of 7.4 (SD 1.7) and eight subjects were controls with an average score of 0.5 (SD 0.9). Seven of the 8 hypermobile subjects demonstrated lower limb hypermobilty (hyperextended knee joints); none of the control subjects had lower limb hypermobility. None of the subjects were seeing a rheumatologist or other specialist for their joints and none of the subjects reported joint pain at the time of testing.

### 2.3 Materials

Surface electromyography (EMG) was used to record muscle activity. Six Iso-dam™ isolated biological amplifiers were used (Model ISO-DAM-B) with VIASYS Healthcare™ silver/silver chloride disposable self-adhesive electrodes. The signals were amplified (×1000) and filtered from 10 Hz to 1 kHz. Data was sampled at 2 kHz using a 1401Plus analogue to digital converter and recorded using Spike2 software (Cambridge Electronic Design UK, version 5.29).

### 2.4 Procedure

The subjects attended a single laboratory session, and written informed consent was provided. Age, sex, height, weight and BMI were recorded. Leg dominance was determined using a modified version of a test outlined in Vauhnik et al. (2008) by asking the following questions; i) which leg would you kick a football with ii) which leg would you squash a bug with and iii) asking the subject to draw a diamond in the air with their foot. The dominant leg was regarded as the one that was used for two or more of the three tasks.

Surface EMG electrodes were placed on the gluteus medius (GM), rectus femoris (RF), semitendinosus (ST), tibialis anterior (TA) and gastrocnemius lateralis (GL) muscles of the dominant leg, and the ipsilateral erector spinae (ES) (Hermens et al., 1999). Briefly, GM was positioned 50% on the line from the iliac crest to the trochanter; RF 50% on the line from the anterior superior iliac spine to the superior part of the patella; ST 50% on the line between the ischial tuberosity and the medial condyle of the tibia; TA one third on the line between the tip of the head of fibula and the tip of the medial malleolus; GL one third on the line between the head of the fibula and the heel and; ES one finger width medial from the line from the posterior superior iliac spine superior to the lowest point of the lower rib, at the level of L2. Two ground electrodes were attached to the ulnar styloid process. Prior to electrode placement, the skin was cleaned with alcohol wipes and allowed to dry. The electrodes were orientated parallel to the muscle fibres, with an inter-electrode distance of 20 mm, and held in place with surgical tape.

Maximum voluntary contractions (MVCs) were initially carried out for each muscle, as follows i) ES: The subject lay prone on a couch and extended their back, velcro straps resisted the lower legs and shoulders; ii) GM: The subject lay on their non-dominant side and abducted their dominant leg against resistance; iii) RF: The subject sat upright with their knees flexed at 90° with the ankle of the dominant leg restrained from extending, and attempted to extend their knee; iv) ST: in the same position, the ankle of the dominant leg was restrained from flexing, and the subject attempted to flex their knee; v) TA: The subject sat upright with their dominant leg in full extension and the foot restrained from dorsiflexion. The subject attempted to dorsiflex the ankle joint and; vi) GL: The subject stood on their dominant leg and attempted to rise up onto their toes while pressure was applied to their shoulders by the investigator. MVCs were performed for 3–5 s, three times for each muscle with a 10 s rest between efforts. Verbal encouragement was provided.

Subjects then performed the following tasks, each for 30 s; i) quiet standing with eyes open (QS EO); ii) quiet standing with eyes closed (QS EC); iii) one-leg standing with eyes open (OLS EO) and; iv) one-leg standing eyes closed (OLS EC). One-leg standing was performed on the dominant leg. For each task the subject was asked to remain with their feet positioned on specific points marked on the floor and to remain as still as possible for 30 s; the timer was started once the subject had established their balance. If the subject lost their balance during the task (and moved their feet from the specific points), the trial was terminated and restarted until they were able to remain balanced for the full 30 s trial.

### 2.5 Data analysis

For each MVC, the root mean square (RMS) value was calculated over 0.2 s intervals of the raw EMG data, using an automated script in Spike2 software. The greatest 0.2 s interval RMS value from the 3 MVCs was taken.

For each muscle, the RMS of the EMG voltage over 0.2 s intervals was calculated throughout each 30 s task. To allow comparison of muscle activity between subjects this was normalised to the peak RMS value during an MVC for that muscle. The normalised RMS values were averaged, disregarding the first and last 3 s of data. This gave one normalised value per muscle for each task.

Co-contraction of antagonistic muscles (RF-ST and TA-GL) was calculated using Equation (1) (Rudolph et al., 2001).

(1) Co-contraction Index = (lower EMG/higher EMG)∗(lower EMG + higher EMG)

where; lower EMG and higher EMG represent the average normalised RMS value of the agonist and antagonist muscles.

Statistical analysis was performed using SigmaPlot statistical package. Two-way analysis of variance (ANOVA) was used to compare tasks and between the hypermobile and control groups for each muscle. Where data was not normally distributed, a logarithm transformation was used. Post-hoc analysis involved an all pairwise multiple comparison procedure using either the Holm-Sidak method or Tukey Test. A *p*-value of <0.05 was taken as significant.

## 3 Results

**Fig. 1:**
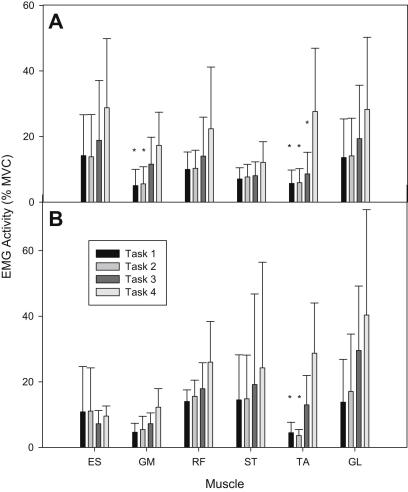
Mean (SD) electromyographic (EMG) activity measured in the erector spinae (ES), gluteus medius (GM), rectus femoris (RF), semitendonosus (ST), tibialis anterior (TA) and gatrocnemius lateralis (GL) muscles for control (A) and BHJS (B) subjects during 30 s each of quiet standing with eyes open (task 1) and eyes closed (task 2), and one-leg standing with eyes open (task 3) and eyes closed (task 4) (∗ denotes significant difference compared with task 4).

All subjects were able to complete each task for 30 s on their first attempt. Fig. 1 shows normalised EMG RMS amplitudes of the 6 muscles measured during the 4 tasks for both groups.

### 3.1 Within group comparisons

ANOVA revealed a significant effect of task on muscle activity (*P* < 0.001). Post-hoc analysis revealed that TA activity was significantly greater during task 4 compared with tasks 1 and 2 for both groups (*P* < 0.001; Fig. 1). GM activity was significantly greater during task 4 compared with tasks 1 and 2 (*P* < 0.05; Fig. 1) within the control group only; although it was observed to increase in the hypermobile group, this did not reach statistical significance.

A co-contraction index was calculated for antagonistic muscles (RF-ST and TA-GL). ANOVA revealed a significant effect of task on TA-GL co-contraction (P < 0.001). Post-hoc analysis revealed that TA-GL co-contraction was significantly more during task 4 compared with 1 and 2 for both groups (*P* < 0.001). ANOVA also revealed a significant effect of task on RF-ST co-contraction (*P* = 0.045). Post-hoc analysis revealed that RF-ST co-contraction increased significantly during task 4 compared with tasks 1 (*P* = 0.008) and 2 (*P* = 0.010) in control subjects only.

### 3.2 Between group comparisons

**Fig. 2:**
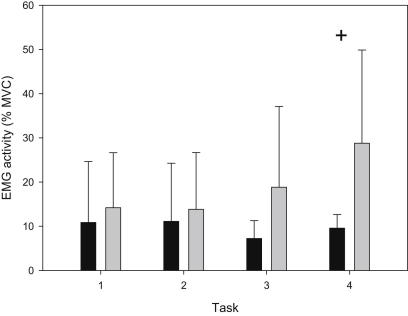
Mean (SD) electromyographic (EMG) activity measured in the erector spinae (ES) of hypermobile syndrome (black bars) and control (grey bars) subjects during 30 s for each of the 4 tasks (+ denotes significant difference between groups).

ANOVA revealed that overall ES activity was significantly more in the control group compared with BHJS group (*P* = 0.019), and post-hoc analysis revealed that ES activity was significantly greater in the control group during task 4 (*P* = 0.017, Fig. 2). ANOVA also revealed that overall ST activity was significantly less in the control group compared with BHJS group (*P* = 0.005). There were no significant differences between groups for the other 4 muscles tested, and there were no significant interactions between group and task for any of the muscles tested.

**Fig. 3:**
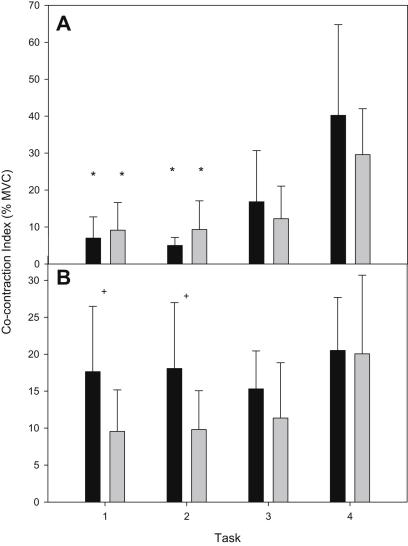
Mean (SD) co-contraction index of tibialis anterior-lateral gastrocnemius (A), and rectus femoris-semitendinosus (B) of BJHS (black bars) and control (grey bars) subjects during 30 s for each of the 4 tasks (∗ denotes significant difference compared with task 4; + denotes significant difference between groups).

There was no significant difference between groups for TA-GL co-contraction (Fig. 3A), however ANOVA revealed a significant effect of group on RF-ST co-contraction (*P* = 0.011). Post-hoc analysis revealed that RF-ST co-contraction index was significantly higher for the BJHS group compared with controls during tasks 1 (*P* = 0.045) and 2 (*P* = 0.041) (Fig. 3B).

## 4 Discussion

This study has demonstrated differences in pelvic and lower limb muscle activation patterns in subjects with pain-free BJHS compared with controls during postural tasks that challenge balance. Both control and BJHS subjects had significantly greater tibialis anterior activity during the more challenging tasks; however only the control subjects had significantly greater gluteus medius activity during these tasks. In addition, control subjects had significantly greater erector spinae activity compared with BJHS subjects during one-leg standing with eyes closed. Hypermobile subjects had significantly higher semitendinosus activation overall, and significantly higher co-contraction of rectus femoris and semitendinosus during the least challenging tasks (two-leg standing).

It has previously been suggested that people use a combination of a “hip strategy” and “ankle strategy”, which generate forces at the hip and ankle joints respectively, to maintain balance during quiet standing and when balance is challenged (Horak and Nashner, 1986; Diener et al., 1988; Runge et al., 1999). In the present study, TA activity increased in both groups as the tasks became more challenging, suggesting an ankle strategy was used by both groups to maintain balance during increased postural sway. However GL activity was only increased in the BJHS group during task 4, perhaps suggesting that the BJHS group relied more heavily on an ankle strategy during the most challenging task.

Gluteus medius is a pelvic stabiliser and acts to abduct the hip joint. The activity of this muscle significantly increased with more difficult tasks, for example during one-leg standing with eyes closed to prevent contralateral pelvic drop and therefore to stabilise the pelvis in control subjects. However in the BJHS group there was no significant increase in GM activity during this task compared with the less challenging tasks, although it did tend to increase suggesting variability in the data, and the difference between the groups did not reach statistical significance (*P* = 0.097). Lower GM activity indicates that some BJHS subjects rely less on the use of a hip strategy to maintain balance during more challenging tasks, as has also been noted in the low back pain population (Mok et al., 2004). This result may have been due to weakness in the GM muscle in BJHS subjects or simply poor motor control patterning; however this was not assessed in the present study. Alternatively, some BJHS subjects may adopt an altered posture whereby they “rest” or “hang” on the hip capsule and hip ligaments rather than activating GM, which would cause pelvic obliquity and instability. The increased ST activity noted in BJHS subjects might be a compensatory mechanism for pelvic instability, as indicated by a correlation between tight hamstrings and lower back pain (Van Wingerden et al., 1997).

Erector spinae activity was similar between groups during the less challenging tasks; similarly no difference in ES activity has been reported in people with and without low back pain during standing (Ahern et al., 1988). However other studies have found increased ES activity in people with chronic low back pain during standing (Alexiev, 1994; Ambroz et al., 2000), and altered ES activity during gait has previously been reported as a direct consequence of low back pain (Lamoth et al., 2006). The only significant difference in ES activity in the current study was noted during the most challenging task (OLS EC), which may indicate differences in lumbopelvic control; however lumbopelvic movement was not measured directly in the present study. Roussel et al. (2009) noted that injury risk in dancers was predicted by lumbopelvic movement control rather than generalised joint hypermobility, thus lumbopelvic control in BJHS requires further investigation.

The BJHS subjects had significantly greater co-contraction of RF and ST than control subjects during less challenging tasks. Control subjects only increased RF-ST co-contraction as a strategy to stabilise the knee during the one-leg standing tasks, thus the BHJS subjects used a strategy during low level tasks that is only used during high level balance tasks in control subjects. Since high levels of co-contraction of antagonistic muscles can increase joint compression (Hodge et al., 1986), the use of this strategy during simple tasks such as quiet standing in the BJHS subjects might put them at higher risk of cartilage degeneration. Greater antagonistic co-contraction, specifically of the quadriceps and hamstrings, has previously been reported in people with knee osteoarthritis during walking (Benedetti et al., 1999; Childs et al., 2004; Lewek et al., 2004; Schmitt and Rudolph, 2007; Hubley-Kozey et al., 2008), perhaps to stabilise a lax knee joint. Lyytinen et al. (2010) reported significantly higher EMG amplitude in vastus medialis in subjects with knee OA compared with control subjects during standing with eyes open and closed, however no co-contraction of quadriceps and hamstrings was found in OA knees during standing in that study (Lyytinen et al., 2010). In the present study, despite the differences in RF-ST co-contraction, there were no significant differences in RF activity between groups.

It is interesting to note that the differences in co-contraction were evident in the less challenging tasks, whereas the differences noted in the pelvic musculature was only evident during the most challenging tasks. This might suggest that the underlying mechanisms are different from one another – RF-ST co-contraction is associated with a necessity to stabilise the knee joint during less challenging tasks in BJHS subjects, whereas poor motor control patterning of the pelvis musculature in BJHS is only evident during more challenging tasks such as OLS, where the base of support is removed.

### 4.1 Clinical relevance

The results presented in this study provide some explanation for the increased risk of developing certain conditions in individuals with BJHS: pelvic instability due to less GM and ES activity during tasks that challenge balance might contribute to lower back pain. In addition, increased co-contraction of the RF and ST might increase compression at the knee joint increasing risk of osteoarthritis at this joint. Currently management of hypermobility is limited until pain or injuries occur, however these findings could be useful with respect to the development of preventative training programs for the BJHS population. Such programs could be developed to correct the altered muscle activity, and to optimise and raise awareness of posture and pelvic stability. This study suggests that key muscle groups for such therapies should include the erector spinae and gluteus medius.

### 4.2 Limitations and future recommendations

The main limitation of this study was low subject numbers and the fact that the two groups were not gender and age matched, thus some non-significant results could be due to the low statistical power of the study or due to age, gender or body mass differences between the subjects in each group. A further limitation was that equipment restraints prevented investigation of additional muscles involved in postural control. Given the lack of previous research in this area, this study focussed on muscles that have been suggested as important in the development of knee OA. The results of the current study suggest that pelvic control may be important in BJHS and therefore it is recommended that future studies investigate this topic further using larger subject numbers and investigating additional muscles involved in postural control (e.g. multifidus, gluteus maximus and tensor fascia latae). The use of motion analysis to monitor specifically pelvis position and movement is also recommended for future work.

## 5 Conclusions

This study has noted differences in muscle activation patterns between pain-free hypermobile and control subjects, specifically involving muscles surrounding the pelvis. Less activity in ES and greater activity of ST as well as RF-ST co-contraction might increase the risk of certain clinical conditions for hypermobile individuals. This study could inform new treatments or preventative strategies for BJHS subjects, and also highlights the relevance of considering the trunk and lower limbs as a dynamic structure rather than considering each joint in isolation.
